# Early onset gastrointestinal cancers - a needs analysis

**DOI:** 10.3389/fonc.2026.1870123

**Published:** 2026-08-13

**Authors:** Nicola Keohane, Oana Deac, John Reynolds, Jessie Elliott, Ravi Narayanasamy, Claire Donohoe, David Gallagher, Nikolett Warner, Cathy Enright, Emily Smyth, John Larkin, Paul McCormick, Michael E. Kelly, Emily Harrold, Maeve Lowery

**Affiliations:** 1 Trinity St James’s Cancer Institute, Dublin, Ireland; 2 Department of Surgery, St James Hospital, Dublin, Ireland; 3 Discipline of Surgery, Trinity College Dublin, Dublin, Ireland; 4 Irish Cancer Society, Dublin, Ireland; 5 School of Medicine, Trinity College Dublin, Dublin, Ireland

**Keywords:** dedicated service, early onset, gastrointestinal cancers, needs analysis, support

## Abstract

**Background:**

Early onset cancer is defined as cancer in patients < 50 years of age. The global incidence of Early Onset (EO) is increasing for unknown reasons. EO Gastrointestinal (GI) cancers, which account for approximately 27% of newly diagnosed cancer cases and 37% of cancer deaths, present distinct challenges in areas including sexual health, finance, career and family. We aimed to assess the holistic needs of these patients attending St James’s Hospital Dublin, a national upper GI referral centre and Ireland’s first OECI Comprehensive Cancer Centre, through a mixed methods approach.

**Methods:**

The primary aim of the needs analysis study was to elicit experiences of patients with EO GI malignancies diagnosed between 2014–2024 attending a single institution. Methods included an anonymous survey and Focus Group Discussions (FGD). 236 surveys were sent to patient’s homes. The survey encompassed 21 domains addressing experiences with guidance and support pertaining to sexual health and function, psychosocial and financial challenges, as well as implications of diagnosis for career trajectory and reintegration into the workforce. Patients were also questioned re preferred timing of referral for speciality services. FGDs used nominal group technique to deepen the analysis.

**Results:**

102 participants responded via survey and 10 participated in an FGD. The mean age at diagnosis was 43 years. 84% of participants reported they would have benefitted from a conversation about fertility, sexual health/function (81%) and financial supports (81%) at diagnosis. 93% of patients were out of work during cancer treatment with average length of time >1 year. 65% were diagnosed late at stages 3 and 4. On average it took 3–6 months before a diagnosis was made and took 2 visits to their GP before speciality referral. 82% did not receive a dedicated referral for care giver supports, with 18% independently engaged in care giver support services.

**Conclusion:**

This needs analysis highlights the importance of specialised clinical pathways, for early onset GI cancer patients focusing on these unique and complex needs. Financial supports, conversations regarding sexual health/function, fertility preservation and psychosocial support are critical areas requiring structured intervention.

## Introduction

The epidemiological profile of cancer incidence is shifting. While advancing age remains the predominant and non-modifiable risk factor, the incidence of early-onset cancers, defined as diagnoses in individuals under 50, is steadily increasing at a rate of approximately 1–2% per year (1). Gastrointestinal (GI) malignancies are rising most rapidly within this group, with colorectal cancer showing the fastest growth with notable contributions from pancreatic, gastric, and oesophageal cancers. Over the past 30 years, the incidence of Early Onset Colorectal Cancer (EOCRC) in the Republic of Ireland has increased by 34%, while the incidence of Late Onset Colorectal cancer (LOCRC) has declined by 12% (2). Between 1994 and 2021, there were 61,180 cases of colorectal cancer among adults older than 20 years in the Republic of Ireland (2). Notably, 60% of EOCRC cases are diagnosed at an advanced stage (stage III or IV). Similarly, early-onset oesophageal adenocarcinoma is rising at a rate of approximately 2% per year (3). However early onset gastric adenocarcinoma cases are stable while cases in patients over 50 years at diagnosis are steadily declining (3). The precise drivers of this trend remain unclear, though it is hypothesised to arise from a complex interplay of modifiable and non-modifiable risk factors.

Early-onset cancer affects patients during their peak productive years. This creates a wide spectrum of psychological and clinical challenges, including disruptions to family life, parenting, relationships, fertility, sexual health, education, career trajectories, financial stability, and overall quality of life. These challenges represent a unique and distinct set of needs for younger cancer patients with younger survivors requiring enhanced support services, dedicated resources and care models that are specifically designed for their age and life stage (4). These patients may have work, education, and/or caregiving responsibilities, and services need to be adaptable and offer flexibility while still meeting their complex needs. Optimally a holistic model of care both provides excellent medical treatment and address patients’ nonmedical needs by providing informational support, social support and integrating patient focused research to improve outcomes (4).

Younger patients are often diagnosed at more advanced stages of cancer and may experience aggressive disease courses, leading to long term consequences (5). These long-term health implications represent an increasing demand on established cancer and diagnostic services (6). Furthermore the later stage of diagnosis associated with early onset cancer often requires resource intensive treatment (7). With treatment costs incurred over a longer lifetime, this can result in higher cumulative expenditures for healthcare services, highlighting the growing burden of early-onset cancer, its significant public health implications, and the urgent need to evaluate and adapt service provision to reflect changing epidemiological patterns (8). The primary aim of this study was to conduct a comprehensive service needs analysis for early onset cancer patients prior to the establishment of a dedicated clinical service. Study specific objectives were to explore patient-perceived optimal level of supportive care services, identify challenges to implementation of services and explore patient experience.

## Methods

This study utilised a mixed methods design to identify the unmet needs of early onset cancer patients. Quantitative data was collected using a cross-sectional anonymous questionnaire and qualitative data was collected through focus groups. Ethics was granted by the Joint Research Ethics Committee (JREC; Project ID: 0278) in St James’s Hospital/Tallaght University Hospital.

### Participants

Early onset upper gastrointestinal and lower gastrointestinal cancer patients who attended Trinity St James’s Cancer Institute (TSJCI) for treatment between 2014–2024 were invited to participate. Participants were eligible if they were diagnosed between the ages of 25-50, were currently alive, had a GI cancer diagnosis and received cancer treatment. Participants were excluded if they were diagnosed before the age of 25 or after age 50, did not meet the criteria for an invasive cancer diagnosis, i.e., were on surveillance for high grade dysplasia, or those who were deceased. Patients under 50 years of age were identified from a prospectively maintained institutional cancer registry. Participants were invited to participate in the anonymous questionnaire via post. Focus group participants were recruited from those who responded to the questionnaire. Written informed consent was obtained from all participants prior to their participation in the focus group.

### Data collection tools

The needs assessment was conducted using a 21-item questionnaire, and two Likert scale questionnaires. The questionnaire was developed by the research team in collaboration with clinical representatives including surgeons, oncologists, psycho-oncologists, and patient representatives. The questionnaire was designed to gather comprehensive insights across two key areas: ‘Diagnostic Experience and Early Supportive Needs’ and ‘Access to Supportive Services and Post-Diagnosis Needs’. Additional information on symptom burden was obtained through eight Likert-scale questions, which explored the extent of these needs in greater depth at specific timepoints along the cancer continuum. Focus group discussion points were informed by the survey findings and developed using the same approach. The focus group interview guide was piloted with public and patient involvement (PPI) representatives. The interview guide addressed areas including financial concerns, sexual health, fertility and survivorship care, follow up services, and service timing. The focus groups were completed in person in TSJCI.

### Data analysis

Quantitative data was analysed using IMB SPSS 26. Distribution was assessed using visual analysis of Q-Q plots and histograms. Data were analysed using descriptive statistics and summarised as frequencies and percentages for categorical variables and means ± standard deviations or and interquartile range for continuous variables.

Qualitative data was analysed using a Nominal Group Technique. Nominal Group Technique is a structured group process that combines individual idea generation with group discussions and voting to efficiently generate and prioritise ideas, preventing domination of a few voices and ensuring all participants contribute equally. Using a Nominal Group Technique (NGT) we systematically identified and prioritised the unmet needs of early-onset cancer patients. Needs were ranked depending on patients’ priority in the areas of fertility, sexual health, financial concerns and follow up care.

Step by step process of Nominal Group Technique - Programme Manager and Patient and Public Partnership Lead introduced the purpose of technique and clarified roles. The next step was silent idea generation; each participant wrote in private to ensure independent thinking. Everyone then contributed their idea in a group, ideas were written down by facilitator without discussion, the group discussed and clarified each idea to ensure correct understanding. Duplicates were eliminated. Participants ranked or voted their ideas and scores were tallied to determine the preferred idea. The highest totalling idea reflected group consensus.

Two focus groups were conducted using the Nominal Group Technique (NGT), each lasting approximately sixty minutes. A total of 10 participants took part. Participant characteristics and participant quotes are summarised in **Supplementary Table 11**. Following discussion of unmet needs identified in the survey, participants independently ranked and prioritised these needs according to perceived importance. The NGT findings were subsequently used to validate and prioritise needs identified through the survey data.

## Results

### Participant characteristics

**Figure 1:**
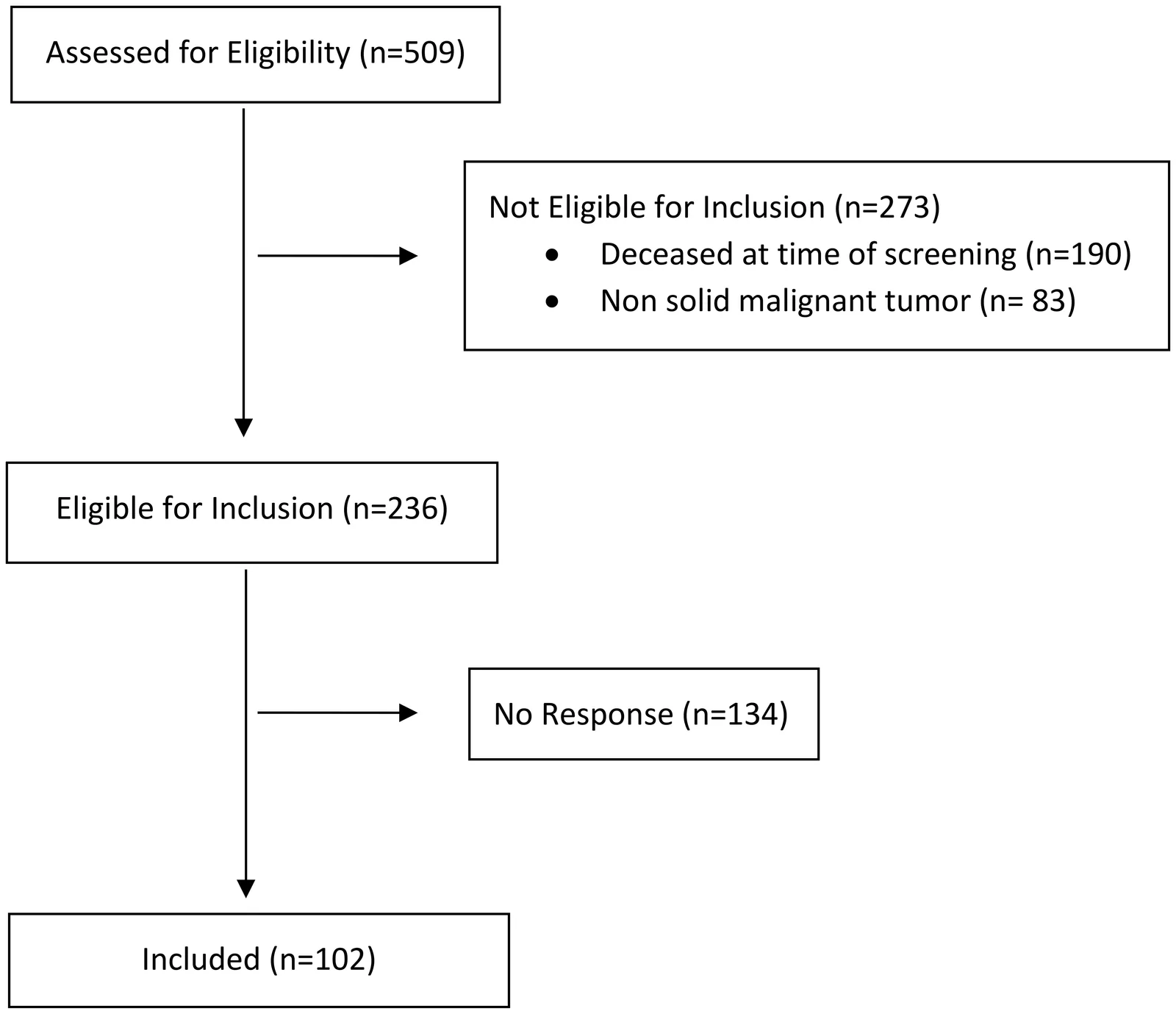
Flow diagram.

Between May 2024 and June 2024, 509 patients managed at St James’s Hospital were identified via the prospectively maintained GI registry, 273 were ineligible for inclusion due to age (<25, >50), invasive malignancy not confirmed histologically or deceased. See **Figure 1**: Flow diagram.

236 eligible patients were invited to participate via postal survey; response rate was 43.2% (102/236) The reasons for non-participation were not provided. In total 102 participants completed the needs analysis questionnaire, and 10 participants took part in the focus groups. Of the 102 participants the mean age was 43, 68% Colorectal, 31% upper GI, with 82% of participant respondents living well and beyond cancer. See **Table 1**: Demographics.

**Table 1:** Demographics.

Total sample	Survey respondents (*n* = 102)	Focus group (*n* = 10)
Age (years)	43.3 (5.3)	44
Cancer type		
Colorectal	69 (68%)	6 (60%)
UGI	33 (32%)	4 (40%)
Stage of cancer journey		
Diagnosis	1 (1%)	0
During treatment	17 (16.8%)	0
Living well with and beyond cancer	84 (82.2%)	10 (100%)
Primary treatment regimen		
Chemotherapy	4 (4%)	0
Surgery	30 (29.7%)	4 (40%)
Chemoradiation	15 (14.9%)	2 (20%)
Chemotherapy & surgery	26 (25.7%)	4 (40%)
Chemoradiation & surgery	25 (24.8%)	0
Radiotherapy & surgery	1 (0.9%)	0

Data is expressed as frequency (%) or mean (SD).

## Cross-sectional survey results

### Diagnostic experience and early supportive needs

Participants reported a median of 4.5 months from symptom onset until presentation for medical review and a further 4.5 months from initial medical review to receiving a formal diagnosis. Prior to referral to a cancer centre, respondents attended a median of two General Practitioner (GP) appointments. Nearly half of respondents, (n=45, 46%) believed that their diagnosis was delayed. Twenty patients (20%) reported receiving fertility counselling. See **Table 2**: Diagnostic experience and early supportive needs.

**Table 2:** Diagnostic experience and early supportive needs.

Diagnostic experience and early supportive needs	Survey respondents (n=102)
Duration of symptoms prior to medical review (months)	4.5 [6]
Duration of time following medical review to formal diagnosis	4.5 [0]
Number of GP appointments prior to referral to cancer centre	2 [2]
Number perceiving delayed diagnosis	45 (44.6%)
Data is expressed as frequency (%) or median [interquartile range]	

### Access to supportive services and post-diagnosis needs

Across the cancer care pathway, participants reported variable access to supportive services, alongside a consistently high perceived need for counselling and support. Fertility counselling was received by 20% of patients; however, 84% indicated a strong preference for this discussion to occur at the time of diagnosis. A similar pattern was observed for sexual health and function counselling, with limited provision (20%) despite a clear preference for early, diagnostic-stage discussions (81%).

Financial counselling and support were also infrequently provided. While only a small proportion of participants reported receiving financial advice (20.8%), the majority expressed a desire for this support, particularly at diagnosis (81.4%). These findings suggest that financial concerns are insufficiently addressed despite being recognised as an important component of care. Caregiver support referrals were also uncommon, with fewer than one fifth of participants (18%) reporting that caregiver support was offered or provided. Where community-based supports were accessed, the Irish Cancer Society was the most frequently utilised resource, while almost half of participants did not engage with any external support services. Engagement with allied health services varied considerably. Physiotherapy was the most commonly accessed service (89.5%), followed by dietetic input (55.8%). Fewer participants accessed social work or psychological supports, while attendance at cancer support groups was reported by just over one fifth. Occupational therapy and speech and language therapy were the least accessed services. Overall, these findings highlight substantial gaps between supportive care needs and service provision across multiple domains, particularly in relation to fertility, sexual health, financial guidance, and caregiver support. See **Table 3**: Access to supportive services and post-diagnosis needs.

**Table 3:** Access to supportive services and post-diagnosis needs.

Support services accessed	Survey respondents (n=102)
Fertility counselling	n= 20 (19.8%)
Sexual health and function counselling	n= 30 (29.7%)
Financial counselling	n= 21 (20.8%)
Physiotherapist	n= 77 (89.5%)
Social worker	n= 27 (31.4%)
Psychologist	n= 18 (20.9%)
Cancer support group	n= 19 (22%)
Dietician	n= 48 (55.8%)
Occupational therapist	n= 14 (16.3%)
Speech and language therapist	n= 12 (14%)
Engagement with external support services	
Referred to the Irish cancer society	n= 24 (27.9%)
Referred to multiple community supports	n= 7 (8.1%)
No referral to cancer support services Caregiver support referral	n= 38 (44%) n= 18 (18.4%)
Perceived benefit of service across timepoints	
Fertility counselling services	
At diagnosis	n= 58 (84.1%)
During treatment	n= 11 (15.9%)
After treatment	n= 0 (0%)
Sexual health and function counselling services	
At diagnosis	n= 57 (76.0%)
During treatment	n= 13 (17%)
After treatment	n= 5 (6.7%)
Financial counselling services	
At diagnosis	n= 70 (81.4%)
During treatment	n= 15 (17.4%)
After treatment	n= 1 (1.25%)

Data is expressed as frequency (%) or mean (SD), median [interquartile range].

### Symptom burden

**Figure 2:**
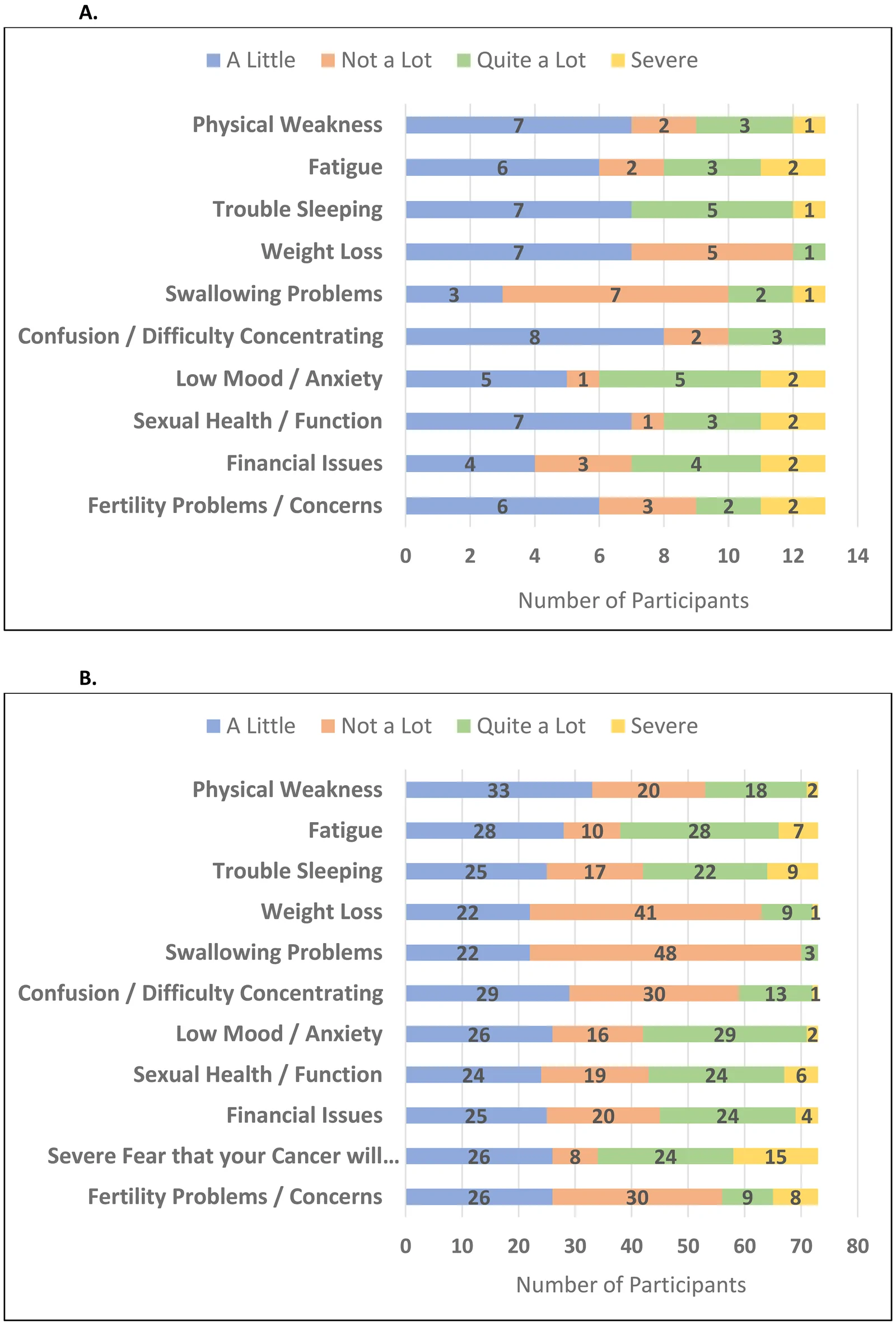
Participant reported symptom severity using a Likert scale (a little to severe). **(A)** Symptom burden between diagnosis and end of treatment. **(B)** Symptom burden after completion of treatment.

Of the 102 participants, 13 participants who completed the Likert scale were between diagnosis and treatment. Symptoms were reported across physical, psychological, sexual, and financial domains from diagnosis through the end of treatment. **Figure 2** illustrates data outlining Likert scale symptom burden based on severity. See **Figure 2A**: Symptom Burden Between Diagnosis and End of Treatment. Physical symptoms such as weakness and fatigue were most frequently rated as “quite a lot” or ‘‘severe’’, whereas weight loss, swallowing difficulties, and sleep disturbances were generally rated as less severe. Psychological, sexual health, and financial concerns were also prevalent. Between diagnosis and the end of treatment, 7 of 13 respondents (53.8%) rated low mood/anxiety as “quite a lot” or “severe”, while 3 of 13 respondents (23.1%) rated difficulties with concentration/confusion as “quite a lot” or “severe’ ‘Sexual health and function concerns were experienced by the majority, and financial strain was noted by two-thirds of participants, with some rating this as severe. Fertility concerns were also reported, with nearly half experiencing at least a little worry, and a minority reporting high levels of concern.

Of the 102 participants, 73 participants who completed the Likert scale were post-treatment and reported a wide range of ongoing concerns. See **Figure 2B**: Symptom Burden after completion of treatment. Physical symptoms remained highly prevalent, with fatigue, physical weakness, sleep disturbance, and residual effects such as weight changes and swallowing difficulties commonly reported. Many participants experienced these symptoms to a moderate or severe degree, indicating that physical recovery after treatment is often incomplete. Cognitive difficulties, including confusion and problems concentrating, also persisted for a substantial proportion of respondents. Psychological, sexual, fertility, and financial concerns were similarly widespread in this post-treatment period. Low mood and anxiety were reported by the majority, and sexual health issues affected nearly two-thirds of participants. Financial issues persisted beyond treatment, with 28 of 73 respondents (38.4%) rating their financial strain as quite a lot or severe. Although fertility concerns varied in severity, 21.9% (16/73) of respondents reported a high level of concern as quite a lot or severe. Notably, fear of cancer recurrence was a significant burden, with more than half experiencing considerable or severe fear. See **Figure 2B**: Symptoms burden after completion of treatment.

### Access to support services

While referrals to physiotherapy (89.5%) and dietetic services (55.8%) were common, uptake of other supportive services was limited, with only 20.9% referred to psychology, 31.4% to social work, 16.3% to occupational therapy, and 14.0% to speech and language therapy. Furthermore, only 18.4% of participants were offered caregiver support referrals, and 44.0% reported no engagement with external patient support services.

## Nominal group technique output

After consolidation of overlapping items, four unmet needs statements were identified. Financial concerns were ranked as highest unmet need (90% rating as high priority), followed by survivorship care (70%), Sexual health (60%) and fertility (50%). In **Table 4**: Unmet Needs Identified (n=10), the total score represents number of participants rating an unmet need as high priority. Ranking is based on total score (participants) not percentage. Of note, ranking is based on Nominal Group Technique (NGT) total scores. The percentage of participants rating each item as a high priority is presented for descriptive context and does not determine ranking order.

**Table 4:** Unmet needs identified (n=10).

Rank	Unmet need identified	Total score	% of participants scoring as high priority
1	Financial concerns	9	90%
2	Survivorship/follow up care	7	70%
3	Sexual health and function counselling	6	60%
4	Fertility counselling	5	50%

## Discussion

This study highlights key unmet needs among younger adults with GI cancer across the diagnostic, treatment, and survivorship continuum. Almost half of respondents (44.6%) perceived a delay in diagnosis, with a median of 4.5 months of experiencing symptoms prior to presenting for medical review, and a further median of 4.5 months from initial review to formal diagnosis. Participants also reported a median of two General Practitioner (GP) consultations before being referred to a specialist cancer centre, underscoring significant pre-diagnostic barriers. These findings align with existing evidence suggesting that younger adults often experience diagnostic delays due to atypical symptom attribution, lower clinical suspicion, and barriers to timely diagnosis (5).

Service-related needs were also identified. Although fertility preservation is recognised as a critical component of care for younger patients, very few reported receiving fertility counselling, and the majority those who did not indicating that such counselling would have been beneficial. The majority of participants identified this as a need during their care. These findings align with study by Keller et al. (9) which reported that only half of early-onset patients were informed of their fertility options. The variability of inclusion in fertility counselling is further illustrated by a number of additional studies. The ASCO Fertility Preservation Guidelines advise that healthcare providers offer a referral, or present reproductive health options, to all patients of reproductive age (10). At Trinity St James’s Comprehensive Cancer Centre, we advocate for an opt-out rather than opt-in model for patients under 50, ensuring that reproductive health counselling is routinely integrated before, during, and after cancer treatment. The importance of this approach is further supported by Chadwick et al. (11), who reported that among 143 patients attending their cancer centre, only 12.6% had a reproductive history recorded at their initial consultation, and just over half engaged in a fertility preservation discussion with their oncologist (11). These findings are consistent with Frederick et al. (12), who similarly noted that early-onset patients often do not receive adequate information or timely referral to reproductive health specialists before commencing cancer therapy (12). Collectively, the evidence underscores a clear and urgent need for early fertility counselling and referral to specialist services prior to treatment initiation (10, 13). Similarly, only very few respondents reported receiving counselling related to sexual health and functioning, despite 79% of non-recipients indicating that such support would have been beneficial.

Analysis of the qualitative data showed that 60% of patients identified this as a high-priority need, while 32.9% reported experiencing ‘quite a lot’ of severity in relation to this need. These gaps are particularly concerning given the long-term impact of cancer treatment on intimacy and sexual wellbeing in younger adults. Acquati et al. (2017) further reinforce this need, with more than half of young adult participants (n = 123) identifying sexual dysfunction as a significant concern within the first months following diagnosis (14). Notably, the study concluded that patients often continue to experience persistent problems with sexual function during the first two years after cancer diagnosis, underscoring the importance of early, proactive, and sustained sexual health support for this population. A systematic review demonstrated that younger cancer patients experience a range of sexual health challenges, and qualitative research has identified system level barriers-such as lack of information, insufficient clinician engagement, and limited access to specialised support that influence whether younger cancer patients receive appropriate sexual and reproductive healthcare (15). These findings underscore a critical and persistent gap in care, highlighting the imperative need to establish a dedicated sexual health and function service capable of addressing the complex age specific needs of younger adults with cancer.

Financial counselling emerged as another area of significant unmet need. Only 20.8% of participants received financial guidance, yet nearly 81% of those who did not receive this support felt it would have been helpful. In the qualitative data, 90% of patients ranked this as their number 1 priority, with 30.8% reporting quite a lot in terms of severity in the between diagnosis and end of treatment. As financial toxicity is increasingly recognised as a key component of cancer burden in young adults, the low uptake of financial support services suggests shortcomings in service integration and provision. Younger adults tend to experience disproportionately higher levels of financial strain, compared with older (16), due to both the nature of their life stage and the cumulative cost of treatment. This challenge is highlighted in JNCI editorial describing that younger adult face financial toxicity and psychological distress (17) This demonstrates that early onset patients face substantial long lasting economic burden, which can influence treatment decisions, adherence to treatment, psychological wellbeing, personal relationships, and long-term quality of life. There is an urgent need for system level changes and early interventions for financial screening, integrated financial counselling into early onset cancer care and access to social and welfare supports tailored specifically to younger adults. It is important to note that the definition of early-onset is often varied, and some studies include patients from the age of 18 to 39. TSJCI utilises the definition of early-onset of 25-50.

Access to psychosocial and supportive services was variable. Fewer than one in five (18.4%) were offered caregiver support referrals, and a substantial proportion (44%) reported not accessing any external patient support services. While referrals to allied health professionals were more common -particularly to physiotherapy (89.5%) and dietetics (55.8%), other important services such as psychology (20.9%), social work (31.4%), occupational therapy (16.3%), and speech and language therapy (14%) were underutilised. This pattern highlights inconsistencies in multidisciplinary care delivery and suggests that many younger patients may not be receiving a fully holistic assessment of needs throughout treatment and survivorship. This is apparent from the qualitative data highlighting patient’s wishes for robust follow up care and survivorship need. A detailed summary of referrals to and utilisation of supportive care services is provided in **Supplementary Data**.

The timing of counselling services also warrants attention. The majority of fertility, sexual health, and financial counselling occurred at, or near, the time of diagnosis. This pattern highlights inconsistencies in multidisciplinary care delivery and suggests that many younger patients may not be receiving a fully holistic assessment of needs throughout treatment and survivorship. The American Psychological Society (APS) emphasises that oncology care for younger adults should be provided by multiple allied health care professionals, and multimodal rehabilitation programme supports younger adults’ unique functional challenges post treatment along with post-surgery. Younger adults often continue to experience long term physical, psychosocial, and financial consequences beyond the initial diagnostic period, and therefore require support delivered longitudinally across the cancer journey (17).

Our study focuses on a specific cancer population whose needs are not frequently captured in general oncology cohort and identifies patients’ needs to inform the development of future services going forward. The use of patient-reported experiences allows for direct insight into perceived gaps in care. This study represents one of the earliest European investigations to specifically examine the needs of individuals diagnosed with early-onset cancer. Despite growing international awareness of the distinct challenges faced by this cohort, the evidence base beyond the US remains notably limited. We highlight unmet supportive care needs, and identify the need for additional patient focused research to better understand, characterise, and address the experiences of this expanding patient group.

Although the study was conducted across two tumour types (upper and lower GI cancers) within a single institution, this setting provided a highly controlled and well-characterised cohort, enhancing the internal consistency and depth of the findings. Participants were diagnosed over a broad time period requiring some respondents to reflect on experiences that occurred many years before survey completion. This may introduce recall bias therefore findings relating to diagnostic experiences and supportive care may be interpreted with caution. Consequently, participants’ reports may not fully reflect the nature or content of interactions that occurred at the time of diagnosis.

Selection bias represents another potential limitation of this study. Of the 236 eligible patients invited to participate, 102 completed the survey, yielding a response rate of 43.2%. As demographic and clinical characteristics of non-responders were not collected, it was not possible to determine whether participants were representative of the wider eligible population. However, the comprehensive capture of patient perspectives is a notable strength of the study, providing valuable insights into the needs of this population. A subgroup analysis has been completed for Upper and Lower GI. Please see supplementary analysis.

While reliance on self-reported data may introduce recall bias and the single-centre design may constrain wider generalisability, Overall, these findings demonstrate substantial unmet needs among younger cancer patients across diagnostic pathways, fertility and sexual health counselling, financial support, caregiver resources, and broader psychosocial services. Improving early recognition of symptoms, embedding multidisciplinary care pathways, and integrating consistent fertility, sexual health, and financial counselling into routine care may significantly enhance patient experience and long-term outcomes. Future research should explore models for streamlined referral pathways, evaluate the impact of unmet needs on quality of life, and identify interventions tailored to the unique challenges faced by younger adults living with and beyond cancer. They highlight the urgent requirement for integrated, developmentally appropriate survivorship pathways that extend beyond the completion of treatment and address the long-term wellbeing of younger adults living with and beyond cancer.

To address these unmet needs, several targeted interventions should be integrated into early onset cancer pathways. Financial concerns may be mitigated through routine screening for financial toxicity at key points in the care trajectory and through access to trained financial navigators or oncology social workers. Structured financial counselling and collaboration with patient support organisations may further support those experiencing economic strain. Sexual health needs require systematic assessment and access to specialised psychosexual therapy and evidence-based interventions for treatment related dysfunction. Fertility concerns highlight the necessity of automatic, timely referral to reproductive medicine for all patients at risk of treatment related infertility, accompanied by comprehensive counselling before, during, and after treatment.

Psychosocial interventions, including routine screening for anxiety, depression, and fear of recurrence, alongside access to psycho-oncology services, may support emotional wellbeing. Age specific peer support programmes may also reduce social isolation and contribute to improved coping. Multidisciplinary survivorship clinics focused on younger adults may address persistent physical sequelae such as fatigue, physical deconditioning, and cognitive symptoms by providing coordinated access to physiotherapy, occupational therapy, dietetics, psychology, and medical review. Embedding unmet needs screening and streamlined referral pathways into routine oncology practice may ensure that younger adults receive timely, developmentally appropriate support across the cancer continuum.

Overall, these findings highlight the requirement for integrated survivorship models tailored to the unique needs of younger adults living with and beyond cancer. By adopting structured, multidisciplinary approaches that explicitly address financial, sexual, fertility, psychological, and physical concerns, cancer services can substantially improve long-term outcomes and quality of life for this population. These findings identify significant unmet needs and suggest areas where dedicated services and additional specialist resources may be beneficial. Further research is required to develop and evaluate targeted interventions designed to address these needs.

## Conclusion

Previous research has shown that patients with early onset cancers may experience unique challenges, including concerns related to fertility, employment, family responsibilities, and navigating healthcare systems, which can differ from those reported by older adults (4) While comparisons with older patients were beyond the scope of the present study, the unmet needs identified by participants highlight the importance of age-appropriate supportive care. Collectively, the findings suggest that a more tailored approach to service provision may be required to meet the needs of this population. Key areas of unmet need identified in this analysis include financial toxicity, sexual health rehabilitation, and reproductive health. The importance of structured follow-up and survivorship care also emerged consistently across the findings. By prioritising tailored interventions and strategic recruitment of appropriately skilled professionals, healthcare systems can ensure that early-onset patients receive the comprehensive support necessary to improve both outcomes and quality of life.

## Data Availability

The raw data supporting the conclusions of this article will be made available by the authors, without undue reservation.
