# Supplementary material for: Early onset gastrointestinal cancers - a needs analysis
Source: Front Oncol. 2026 Aug 13;16:1870123. doi: 10.3389/fonc.2026.1870123 (PMC13518181; doi:10.3389/fonc.2026.1870123)
Supplement: Supplementary file 1 [file DataSheet1.pdf]

Please see supplementary data below including

-Table 5: Lower GI subgroup analysis

-Table 6: Upper GI subgroup analysis

-Table 6: Supportive care needs Lower GI

-Table 7: Supportive Care needs Upper GI

-Descriptive analysis based on age as requested- 25-35 completed first then 40-50.

-Access to support services

-Characteristics of participants from focus groups and quotes

**Table 5: Lower GI subgroup analysis**	
**Category**	**Finding**
**Average Age at Diagnosis**	43 years
**Sex Distribution**	Female: 51.1%<br>Male: 48.9%
**Tumour Site**	Rectum: 37.6% (most common site)<br>Sigmoid colon: 22.5% (second most common site)<br>Rectum and sigmoid colon combined: 60.1% of cases<br>Anus: 13.6% of cases
**Treatment Intent**	Curative intent: 79.2%<br>Palliative intent: 15.2%
**Clinical Presentation**	PR bleeding: 47.3%<br>Abdominal pain: 32.3%<br>Altered bowel habit: 21.4%<br>Weight loss: 11.8%<br>Perianal discomfort: 10.0%<br>Anaemia: 6.8%

Table 6: Upper GI subgroup analysis	
**Category**	**Finding**
**Average Age at Diagnosis**	43 years
**Sex Distribution**	Male: 72.7%<br>Female: 27.3%
**Tumour Site**	Oesophagogastric (OG) junction: 35.5% most common site<br>Lower oesophagus: 21.2%<br>Stomach: 13.9%
**Clinical Presentation**	Dysphagia: approximately 50–70% of patients<br>Weight loss: approximately 40–60%<br>Reflux/heartburn: approximately 20–40%
**Treatment Intent**	Radical intent: 59.2%<br>Palliative intent: 32.9%<br>Initially radical, transitioned to palliative within 6 months: 4.8%

Survivorship challenges and supportive care needs

Table 7: Lower GI			
**Domain**	**Discussion Occurred (%)**	**No Discussion (%)**	**Would Have Benefited from Discussion (%)**
Fertility	21.4%	78.6%	71.1%
Sexual Health	35.7%	64.3%	80.0%
Financial Support	25.0%	75.0%	93.3%
**Upper GI**			
**Domain**	**Discussion Occurred (%)**	**No Discussion (%)**	**Would Have Benefited from Discussion (%)**
Fertility	20.0%	80.0%	62.5%
Sexual Health	25.0%	75.0%	86.7%
Financial Support	20.0%	80.0%	87.5%

Comment #2

Please see below descriptive subgroup analysis of young onset GI patients from 25-30 then 40-50 in the areas of sexual health, fertility and financial concerns.

Demographic Characteristic	Result
Age Group	25–35 years
Tumour Type	Colorectal Cancer (CRC): 61.5%<br>Upper Gastrointestinal (UGI): 38.5%
Stage of Cancer Journey	Living well and beyond cancer: 100%

Fertility 25-35

Fertility was identified as an important area of supportive care among respondents aged 25–35 years. When asked whether a healthcare professional had spoken to them about fertility, 62.5% reported that no discussion had taken place, while 37.5% stated that fertility had been discussed during their cancer journey. Despite the relatively low frequency of these conversations, most respondents expressed a desire for fertility-related information and support. Specifically, 72.7% reported that they would have found a discussion about fertility helpful, whereas 27.3% indicated that such a discussion would not have been beneficial. These findings suggest that fertility is a significant concern for many adults aged 25–35 years and that opportunities may exist to improve routine fertility-related discussions within cancer care services.

Sexual Health 25-35

Sexual health was another area where respondents reported limited communication with healthcare professionals. Most participants 84.6% stated that a healthcare professional did not no speak to them about sexual health, while only 15.4% recalled having such a discussion. Despite the lack of conversations, the majority of respondents 72.7% indicated that they would have found a discussion about sexual health beneficial, compared with 27.3% who stated that it would not have been beneficial. This finding highlights a potential gap between patients' informational and supportive care needs and the support currently being provided.

Financial 25-35

Financial concerns were the least discussed topic among respondents. Only 7.7% reported that a healthcare professional had spoken to them about the financial impact of cancer, while 92.3% stated that no discussion had taken place. Nevertheless, most respondents 83.3% indicated that they would have found a conversation regarding financial concerns beneficial, whereas 16.7% reported that such a discussion would not have been useful. These findings suggest that financial wellbeing is an important but potentially overlooked aspect of survivorship care for younger adults affected by cancer.

Overall, respondents aged 25–35 years reported relatively low levels of discussion with healthcare professionals regarding fertility, sexual health, and financial concerns. While discussions about fertility occurred slightly more frequently than discussions about sexual health or finances, substantial proportions of respondents indicated that these topics were either not addressed or inadequately explored. Importantly, the majority of respondents reported that conversations about these issues would have been beneficial, suggesting unmet supportive care needs within this younger adult cancer survivor population. The findings indicate opportunities to strengthen holistic survivorship care by incorporating routine assessment and discussion of fertility, sexual health, and financial wellbeing.

Please see subgroup descriptive analysis within the ages of 40-50.

**Demographic Characteristic**	**Result**
**Age Group**	40–50 years
**Stage of Cancer Journey**	Living Well and Beyond: 79.7%
	Currently undergoing active cancer treatment: 20.3%

<u>Fertility 40-50</u>

Discussion of fertility appeared to be relatively uncommon among respondents aged 40–50 years. The majority of participants (87%) reported that a healthcare professional did not speak with them about fertility during their cancer journey, while only 17% recalled having a fertility-related discussion. Despite the lack of fertility conversations, respondents generally viewed fertility information and support as valuable. Three-quarters of participants (75%) indicated that they would have found a discussion about fertility beneficial, compared with 25% who reported that it would not have been helpful.

Sexual Health 40-50

Sexual health was not routinely discussed with respondents aged 40–50 years during their cancer journey. Almost three-quarters of participants (74%) reported that no healthcare professional had spoken to them about sexual health, while only 26% indicated that such a discussion had taken place. Despite the limited number of sexual health discussions, the vast majority of respondents perceived these conversations as valuable. More than four-fifths (83%) reported that they would have found a discussion about sexual health beneficial, whereas 17.7% indicated that it would not have been beneficial. These findings suggest a notable gap between the sexual health information and support provided and the needs of patients in this age group. Although sexual wellbeing is an important aspect of quality of life and survivorship, discussions on this topic appear to occur infrequently. The high proportion of respondents who would have valued a conversation about sexual health highlights the importance of incorporating routine assessment and discussion of sexual wellbeing into cancer care and survivorship services for adults aged 40–50 years.

Financial 40-50

Discussions regarding the financial impact of cancer were uncommon among respondents aged 40–50 years. The majority of participants (79%) reported that no healthcare professional had spoken to them about financial concerns during their cancer journey, while only 20% indicated that they had received support or information relating to financial matters.

Despite the limited frequency of these conversations, respondents overwhelmingly recognised their value. Ninety-two per cent (92%) stated that they would have found a discussion about financial concerns beneficial, compared with 7% who reported that it would not have been helpful.

These findings suggest that financial wellbeing is an important but potentially under-addressed aspect of cancer care for individuals aged 40–50 years. While most respondents did not recall receiving information or support regarding the financial implications of cancer, the vast majority felt that such discussions would have been beneficial. This highlights a potential unmet supportive care need and suggests that routine assessment of financial concerns may help to improve the overall patient experience and support available throughout treatment and survivorship.

Supplementary Table . Referral to and utilisation of psychosocial and supportive care services

Service / Support	Percentage (%)
Physiotherapy referral	89.5
Dietetics referral	55.8
Social work referral	31.4
Psychology referral	20.9
Caregiver support referral	18.4
Occupational therapy referral	16.3
Speech and language therapy referral	14.0
Reported no access to external patient support services	44.0

Focus group characteristics and quotes:

UGI Focus Group	CRC Focus Group
Number of participants: 4	Number of participants: 5
Duration: 1 hour	Duration: 1 hour
Gender: 3 male, 1 female	Gender: 2 male, 3 female
Participant ages: 44, 46, 41 (male); 33 (female)	Participant ages: 43, 41, 36 (female); 43, 40 (male)
Stage of cancer journey: 1 male during treatment; 1 male participating in a clinical trial; 1 male living well and beyond cancer; 1 female during treatment	Stage of cancer journey: 2 females during treatment; 3 participants living well and beyond cancer

Illustrative participant quotations – UGI focus group

"Sexual function issues were something that cropped up after treatment."

"No one really talks about it."

"Having something like this, a forum to discuss and talk about things is great."

"I've never met someone with the same type of cancer as me."

Illustrative participant quotations – CRC focus group

"There was no one to call when I was discharged from care."

"I didn't want to pick up the phone to support services; I wanted them to call me."

“You have lots of questions when you're diagnosed; who do you ask?”

“Young-onset support services should be offered as part of routine care, with the option to opt out.”
